# Supplementary material for: A qualitative exploration of migraine in students attending Irish Universities
Source: PLoS One. 2024 Aug 23;19(8):e0305643. doi: 10.1371/journal.pone.0305643 (PMC11343468; doi:10.1371/journal.pone.0305643)
Supplement: S1 File — Poster used for study recruitment provided via Microsoft Word Document. SI A2. Participant Information Leaflet. Leaflet used to describe study provided via Microsoft Word Document. SI A3. Participant Consent Form. Informed consent questions prior to visiting electronic link provided via Microsoft Word Document. SI A4. Migraine Illustration. Sample of a migraine illustration provided via Microsoft Word Document. SI A5. Focus Group Questions. Focus group questions provided prior to study participation, provided via Microsoft Word Document. SI A6. Standards for Reporting Qualitative Research. Checklist for study rigor, completed via Microsoft Word Document. (DOCX) [file pone.0305643.s002.docx]

## **Table of Contents**

SI A1. Recruitment Poster

SI A2. Participant Information Leaflet

SI A3. Participant Consent Form

SI A4. Migraine Illustration

SI A5. Focus Group Questions

SI A6. Standards for Reporting Qualitative Research Checklist

**Supporting Information A1. Recruitment Poster**

**Participant Recruitment**

**Migraine: The Lived Experience**

**UCD School of Public Health, Physiotherapy and Sports Science**

UCD Ethics Application Number: LS-21-83-Flynn-Fullen

**DO YOU SUFFER FROM MIGRAINE?**

**You are invited to take part in a focus group!**


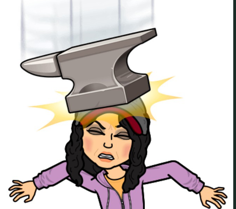
**
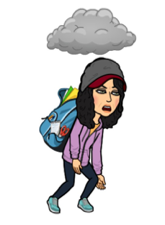

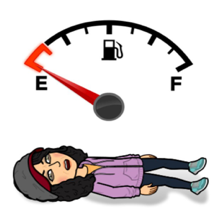
**

This migraine research involves an anonymised one-time audio-only Zoom focus group.

We wish to learn more (i) what triggers your migraine, (ii) what your symptoms are, (iii) how you manage it, (iv) what you know about migraine, and (v) how migraine impacts in various areas of your university, social, family and work life. If you agree to participate, an anonymous questionnaire will be collected. You can also provide a drawing of your migraine experience if you wish. A time will then be arranged for a one-off focus group interview, which will take approximately 60 minutes. Please note that you may be in a focus group where another participant recognises your voice, so may be able to identify you. However, that would be a coincidence and is not the intention but a possibility. Thus, confidentiality cannot be guaranteed concerning the content that will be discussed in the focus group.

Research Team – contact Orla Flynn if you would like further information about the study.

Associate Professor Brona Fullen

UCD School of Public Health, Physiotherapy & Sports Science

University College Dublin, UCD Centre for Translational Pain Research

Brona.fullen@ucd.ie

Research Supervisor

Ms Orla Flynn, PhD Candidate, BSc Physiotherapy. BSc Health & Performance Science

UCD School of Public Health, Physiotherapy & Sports Science, UCD Centre for Translational Pain Research

Orla.flynn.1@ucdconnect.ie

Researcher

You can access more general information on the Migraine Association of Ireland website: [www.migraine.ie](http://www.migraine.ie)

**Supporting Information A2. Participant Information Leaflet**


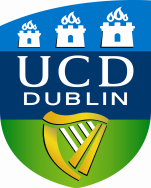


**Participant Information Leaflet**

**Migraine: The Lived Student Experience**

UCD School of Public Health, Physiotherapy and Sports Science

UCD Centre for Translational Pain Research

**UCD Ethics Application Number: LS-21-83-Flynn-Fullen**

**INTRODUCTORY STATEMENT:** My name is Orla Flynn, and I am undertaking a PhD in the UCD School of Public Health, Physiotherapy and Sports Science and with the UCD Centre of Translational Pain Research, examining ***the experience of migraine in university students.***

Migraine is a recurrent throbbing headache that typically affects one side of the head and is often accompanied by nausea, disturbed vision, or sensitivity to senses, including light, sound, or smell. To participate in this study, you must have been clinically diagnosed with migraine by a medical professional and be 18 years of age or older.

**WHAT IS THE RESEARCH ABOUT?**

The research will explore university students' experience of living with migraine by asking you to take part in a focus group with other students who suffer with migraine. You will be asked to answer some questions about yourself and your migraine experience via an anonymous questionnaire administered via Qualtrics. You also have the option to provide an illustration of your migraine experience using a Microsoft Word document, before you take part in the focus group. In the focus group, which will be conducted via Zoom (audio-recorded), you will be asked questions about your understanding of your condition, how your migraine presents, what triggers a migraine and how you manage it. We will also ask how migraine impacts on your university, social, family and work life.

**WHY ARE WE DOING THIS RESEARCH?** Whilst we know that migraine is a significant problem, not much is known about how students manage their migraine and how much it impacts on their daily life. This study will contribute to research in this area, and hopefully help students better manage their migraines. To achieve this aim we will ask you to complete a questionnaire with some information about you and your migraine experience, and then take part in a Zoom focus group (audio-recorded) to further understand your lived experience of migraine, specifically how it presents, what triggers it and how you manage it and how it impacts on your university, social, family and work life. You can also provide a drawing of your migraine experience if you wish.

**WHY HAVE YOU BEEN INVITED TO TAKE PART?** You have been invited to take part as you are a university student diagnosed with migraine.

**HOW WILL MY DATA BE USED?** Information collected during the focus group will be anonymized, written up and submitted for publication as part of my PhD study. All information will be treated in the strictest confidence, and you will not be identifiable in anyway. All information you provide will be encrypted and stored on password protected computers and a USB backup key that will be held securely in the locked offices of the principal investigator Orla Flynn and supervisor Associate Professor Brona Fullen. The data will then be destroyed in line with UCD policy. Under freedom of information legislation, you are entitled to access the information provided at any time while it is in storage as specified above.

**WHAT WILL HAPPEN IF I DECIDE TO TAKE PART IN THIS RESEARCH STUDY?**

If you choose to take part, you will be asked to participate in ***a one-time audio recorded focus group interview conducted via Zoom.*** The focus group with up to six students will take approximately 45-60 minutes. Your identity will always be kept confidential, and you will not be identified in any way, either during the audio-recorded Zoom focus group, or in the research publications. The focus group will be conducted via audio Zoom only so you will not have your camera switched on. You may be in a focus group where another participant may recognise your voice so may be able to identify you, but that would be by coincidence and is not the intention but a possibility. Thus, confidentiality cannot be guaranteed in relation to the content that will be discussed among participants in the focus groups. You will not be identifiable in any research publications that arise from this study.

Before agreeing to take part in the study you will be sent:

(i) a copy of the demographic form that you would complete ahead of the focus group. This form asks for your age, course of study, type of migraine you have, migraine pattern, what your level of migraine knowledge is and what treatments and management strategies you use.

(ii) a copy of the questions you will be asked in the focus group. These questions are to learn more about your understanding of your migraine condition, such as how your migraine presents, what triggers it, how you manage it and the impact it has on your life. We will also include a link to the Migraine Association of Ireland if you would like more general information on migraine.

If you have any questions about the study, please contact Orla Flynn at [orla.flynn.1@ucdconnect.ie](mailto:orla.flynn.1@ucdconnect.ie) and she can answer any questions you may have.

Once you are happy to take part in the study, please re-contact Orla and you will be sent a secure link to an Informed Consent Form to complete and return within 7 days. Once your consent form has been received you will be sent a secure link to complete the demographic questionnaire, (takes approximately 5 minutes to complete). You have up to 7 days to complete and return this form. Next, Orla Flynn will send you a Doodle Poll link with the dates and times of the Zoom focus groups that will allow you to book yourself into one of the focus groups that suits. If none of the times suit, we can offer additional dates and times based on feedback. During the audio-recorded zoom focus group you will be asked the same questions that you will have seen previously.

**HOW WILL MY PRIVACY BE PROTECTED?** All information provided by participants will be treated confidentially. Once you consent to take part you will be assigned a study number for the purposes of identification, and you will be asked to use this number on your Zoom during the focus group. The focus group will be conducted via audio Zoom only so you will not have your camera switched on. You may be in a focus group where another participant may recognise your voice so may be able to identify you, but that would be by coincidence and is not the intention but a possibility. Thus, confidentiality cannot be guaranteed in relation to the content that will be discussed among participants in the focus groups. The focus group will be recorded, and the audio recording will be stored on an encrypted file on a password protected computer. Transcripts of your audio-recorded Zoom focus group will be made with all identifying information removed. Extracts from your focus group may be used anonymously in published research or in conference presentations. However, your personal information or any other information that might identify you will be removed. The study information will be retained for up to 3 years, until the publication potential of the PhD has been maximized and then destroyed in line with UCD policy. You will not be identifiable in any research publications that arise from this study.

**WHAT ARE THE BENEFITS OR RISKS ASSOCIATED WITH THE STUDY?** There are no direct benefits to you. By providing your insight, the study findings, once published, have the potential to contribute to our understanding of how to better the health of students who suffer migraines.

**WHAT ARE THE RISKS OF PARTICIPATING IN THIS RESEARCH?** There is some risk that you may experience emotional distress when discussing the impact of migraine on your daily life. If this happens the focus group will be paused to allow you time to recover and decide to continue or you may prefer to withdraw from the focus group. A breakout room will also be provided where you can take a break and decide if you would like to continue with the study. One of the facilitators will join you to support you. The researchers asking the questions are experienced clinicians and can also direct you to student health services <https://www.ucd.ie/studentadvisers/about/staffprofiles/>

You are also encouraged to speak to your GP or migraine care provider if participating in this research has caused you any distress. If you wish to access more information about migraines, please visit the website of The Migraine Association of Ireland: [www.migraine.ie](http://www.migraine.ie).

**CAN I CHANGE MY MIND AT ANY STAGE AND WITHDRAW FROM THE STUDY?** Yes, you can. You can decide to withdraw from the study at any point, during, or up to two weeks after the focus group by contacting Orla Flynn. Your focus group answers can be excluded from the data analysis and your consent form and demographic form will be destroyed in line with UCD policy. This will not in any way affect your academic study in UCD. Once your data has been permanently anonymized (two weeks after the study concludes) you will no longer be able to withdraw from the study. All information collected will be merged and reported as group summary data.

**HOW WILL I FIND OUT WHAT HAPPENS WITH THE PROJECT?** The study will be submitted for publication when it is completed. Please let Orla Flynn know if you would like to be kept informed about the study results.

**NEXT STEPS:** If you are interested in learning more about the study or are willing to take part, please contact the Orla Flynn at the email below:

**RESEARCH TEAM**

Orla Flynn, PhD Candidate, BSc Physiotherapy, BSc Health and Performance Science, PGC Exercise and Nutrition Science, UCD School of Public Health, Physiotherapy and Sports Science, UCD Centre for Translational Pain Research. [Orla.flynn.1@ucdconnect.ie](mailto:Orla.flynn.1@ucdconnect.ie)

Brona Fullen, PhD, MSc, BSc, Associate Professor, UCD School of Public Health, Physiotherapy and Sports Science, UCD Centre for Translational Pain Research. [Brona.fullen@ucd.ie](mailto:Brona.fullen@ucd.ie)

**Supporting Information A3. Participant Consent Form**


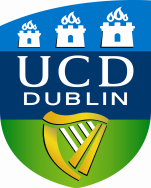


**Migraine: The Lived Experience**

UCD School of Public Health, Physiotherapy and Sports Science

UCD Centre for Translational Pain Research

**UCD Ethics Application Number: LS-21-83-Flynn-Fullen**

***Consent to Participate in Research***

I understand that: I have had the purpose and nature of the study explained to me in writing and I have had the opportunity to ask questions about the study.

*Consent box will be available in Google Forms and states as follows: Yes, I understand, and I consent ☐ No, I do not consent ☐*

I understand that: Even if I agree to participate now, I can withdraw at any time during and up to two weeks after the focus group interview and all my data will be removed and destroyed. After the two-week period the data will be anonymized and analyzed. Once my data has been permanently anonymized, I will no longer be able to withdraw from the study.

*Consent box will be available in Google Forms and states as follows: Yes, I understand, and I consent ☐ No, I do not consent ☐*

I understand that: I can choose not to answer any question if I choose.

*Consent box will be available in Google Forms and states as follows: Yes, I understand, and I consent ☐ No, I do not consent ☐*

I understand that: I am aware that I may find some of the questions in the interview and/or questionnaires upsetting and that I can ask to pause or stop the interview at any time if I feel I cannot continue. I can also go into a separate zoom breakout room to take time and that one of the focus group facilitators will be there to support me.

*Consent box will be available in Google Forms and states as follows: Yes, I understand, and I consent ☐ No, I do not consent ☐*

I understand that: Participation in the study involves me completing a demographic questionnaire about my age, gender, major, college of the university, type of migraine, migraine knowledge and migraine management.

*Consent box will be available in Google Forms and states as follows: Yes, I understand, and I consent ☐ No, I do not consent ☐*

I understand that: I can provide an illustration of my migraine experience via a Microsoft Word document, prior to study commencement. This illustration can be sent to the research team via email. Upon receipt of this illustration, it will be downloaded and encrypted so it is not accessible by anybody but the research team. Any image provided will be securely stored in Associate Professor Brona Fullen’s locked office and used anonymously in any publications that arise from this research study. The illustration will not be linked to you in any way so that your confidentiality is always protected in this regard.

*Consent box will be available in Google Forms and states as follows: Yes, I understand, and I consent ☐ No, I do not consent ☐*

I understand that: Participation in the study involves me taking part in an audio-recorded zoom focus group interview with up to five other students. I will be asked questions that have already been sent to me to review ahead of consenting to participate.

*Consent box will be available in Google Forms and states as follows: Yes, I understand, and I consent ☐ No, I do not consent ☐*

I understand that: Whilst every effort has and will be made to provide anonymity, another participant in the focus group may recognize my voice, so may be able to identify me but that would be by coincidence and is not the intention but a possibility. Thus, confidentiality cannot be guaranteed in relation to the content that will be discussed among participants in the focus groups.

*Consent box will be available in Google Forms and states as follows: Yes, I understand, and I consent ☐ No, I do not consent ☐*

I understand that: I will not benefit directly from participating in this research.

*Consent box will be available in Google Forms and states as follows: Yes, I understand, and I consent ☐ No, I do not consent ☐*

I understand that: All information I provide for this study will be treated confidentially. Anonymized extracts from my interview may be quoted in a PhD thesis for publication or conference presentation but I will not be personally identifiable from either the quantitative or qualitative data and analysis.

*Consent box will be available in Google Forms and states as follows: Yes, I understand, and I consent ☐ No, I do not consent ☐*

I understand that: If I inform the researcher that myself or someone else is at risk of harm, they may have to report this to the relevant authorities - they will discuss this with me first but may be required to report with or without my permission.

*Consent box will be available in Google Forms and states as follows: Yes, I understand, and I consent ☐ No, I do not consent ☐*

I understand that: All study data will be anonymized, encrypted, and stored on both the principal investigator Orla Flynn and Associate Professor Brona Fullen’s’ password protected computers which will be held in locked offices, for three years after the study has finished. I understand this encrypted information will be backed up on a USB key which will also be stored in Associate Professor Brona Fullen’s’ locked office.

*Consent box will be available in Google Forms and states as follows: Yes, I understand, and I consent ☐ No, I do not consent ☐*

I understand that: The data will then be destroyed in line with UCD Data Protection Policy.

*Consent box will be available in Google Forms and states as follows: Yes, I understand, and I consent ☐ No, I do not consent ☐*

I understand that: Under freedom of information legislation, I am entitled to access the information I have provided at any time while it is in storage as specified above.

*Consent box will be available in Google Forms and states as follows: Yes, I understand, and I consent ☐ No, I do not consent ☐*

I understand that: I am free to contact any of the people involved in the research to seek further clarification and information.

*Consent box will be available in Google Forms and states as follows: Yes, I understand, and I consent ☐ No, I do not consent ☐*

I understand that: I voluntarily agree to participate in this research study.

*Consent box will be available in Google Forms and states as follows: Yes, I understand, and I consent ☐ No, I do not consent ☐*

If you are interested in taking part in the study, you are asked to give written informed consent via an online form which has the exact same question format as you have just read. Please click the link below to fill out the online consent form and respond to each question by clicking on the relevant box:

**Google Forms link:**

[***https://docs.google.com/forms/d/e/1FAIpQLSexrzAYsYbbnzks2_qqX3iHYcFtcIgUgkRKlkiymGsGHKcYeQ/viewform?usp=sf_link***](https://docs.google.com/forms/d/e/1FAIpQLSexrzAYsYbbnzks2_qqX3iHYcFtcIgUgkRKlkiymGsGHKcYeQ/viewform?usp=sf_link)

*Thank you.*

Brona Fullen

Associate Professor

UCD School of Public Health, Physiotherapy & Sports Science

UCD Centre for Translational Pain Research

University College Dublin

Brona.fullen@ucd.ie

01 7166516

Orla Flynn

PhD Candidate

UCD School of Public Health, Physiotherapy & Sports Science

UCD Centre for Translational Pain Research

Orla.flynn.1@ucdconnect.ie

**Supporting Information A4. Migraine Illustration**


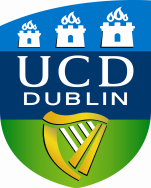


**Illustration “What migraine means to me”**

If you would like to illustrate what migraine means to you, please use the “draw” function and “insert drawing canvas” in Microsoft Word to draw a picture below. You can also color this if you wish.

**Supporting Information A5. Focus Group Questions**

| 1. Migraine Knowledge  - What is your understanding of what a migraine is? - Who/where do you trust to get information about your migraine from, and how do you verify the sources? |
| --- |
| 1. Migraine Characteristics  - How do you know a migraine is coming on? |
| 1. Migraine Triggers  - Do you think lifestyle factors trigger your migraines – for example sleep, exercise, eating, stress? |
| 1. Migraine Treatments   Migraine prevention   - If you think a migraine is coming on, is there anything you can do to prevent it developing further? - How does using preventative treatment impact your day? - For example, does preventing a migraine take extra time out of your day? - Are there any methods for preventing migraine that you are aware of but haven’t tried, if so, why?   During a migraine attack   - How do you manage a migraine attack? - What other strategies do you find useful? For example - exercise, massage, hot baths, - herbal preparations, or dietary supplements? - Do any of these have side effects? - Are there any methods for treating migraine that you are aware of but haven’t tried, if - so, why? |
| vi. Medical Management   - Do you regularly attend a doctor or clinician for your migraine? - Do you feel your clinician provides enough information about your - condition? - Are there other treatments you would like to know more about? - Tell us the best way your doctor could provide you with migraine information. For - example, through leaflets, videos, or websites? - What would you say a “good consultation” with your migraine specialist looks like? - When you have a medical appointment, do you feel listened to? - Are you overall satisfied with how your migraine is managed? - Do you see any other healthcare professionals to help you manage your migraine, if so, who? |
| vii. Migraine Impact:   - How does a migraine affect your everyday life? For example, university, social life, - family life, and work (if you have a job outside university). - What is one phrase you would use to describe your migraine? - What would it mean to you to not have migraine anymore? |
| viii. Other comments   - Are there any other comments you would like to make about your overall migraine experience? |

- Questions were iterative; further questions could be generated from participant answers.

**SI FILE A6. Standards for Reporting Qualitative Research Checklist**

<http://www.equator-network.org/reporting-guidelines/srqr/>

|  | **Section** | Page |
| --- | --- | --- |
|  | **Title** - Concise description of the nature and topic of the study identifying the study as qualitative or indicating the approach (e.g., ethnography, grounded theory) or data collection methods (e.g., interview, focus group) is recommended | 1 |
|  | **Abstract** - Summary of key elements of the study using the abstract format of the intended publication; typically includes background, purpose, methods, results, and conclusions | 2 |
|  |  |  |
| **Introduction** | |  |
|  | **Problem formulation** - Description and significance of the problem/phenomenon studied; review of relevant theory and empirical work; problem statement | 3 |
|  | **Purpose or research questio**n - Purpose of the study and specific objectives or questions | 4 |
|  |  |  |
| **Methods** | |  |
|  | **Qualitative approach and research paradigm** - Qualitative approach (e.g., ethnography, grounded theory, case study, phenomenology, narrative research) and guiding theory if appropriate; identifying the research paradigm (e.g., postpositivist, constructivist/ interpretivist) is also recommended; rationale** | 10-11 |
|  | **Researcher characteristics and reflexivity** - Researchers’ characteristics that may influence the research, including personal attributes, qualifications/experience, relationship with participants, assumptions, and/or presuppositions; potential or actual interaction between researchers’ characteristics and the research questions, approach, methods, results, and/or transferability | 13 |
|  | **Context** - Setting/site and salient contextual factors; rationale** | 4-11 |
|  | **Sampling strategy** - How and why research participants, documents, or events were selected; criteria for deciding when no further sampling was necessary (e.g., sampling saturation); rationale** | 6-7 |
|  | **Ethical issues pertaining to human subjects** - Documentation of approval by an appropriate ethics review board and participant consent, or explanation for lack thereof; other confidentiality and data security issues | 4 |
|  | **Data collection methods** - Types of data collected; details of data collection procedures including (as appropriate) start and stop dates of data collection and analysis, iterative process, triangulation of sources/methods, and modification of procedures in response to evolving study findings; rationale** | 8-11 |
|  | **Data collection instruments and technologies** - Description of instruments (e.g., interview guides, questionnaires) and devices (e.g., audio recorders) used for data collection; if/how the instrument(s) changed over the course of the study | 8-11; SI A; SI B |
|  | **Units of study** - Number and relevant characteristics of participants, documents, or events included in the study; level of participation (could be reported in results) | 14-15, SI B |
|  | **Data processing** - Methods for processing data prior to and during analysis, including transcription, data entry, data management and security, verification of data integrity, data coding, and anonymization/de-identification of excerpts | 11-14 |
|  | **Data analysis** - Process by which inferences, themes, etc., were identified and developed, including the researchers involved in data analysis; usually references a specific paradigm or approach; rationale** | 11-14 |
|  | **Techniques to enhance trustworthiness** - Techniques to enhance trustworthiness and credibility of data analysis (e.g., member checking, audit trail, triangulation); rationale** | 11-14 |
|  |  |  |
| **Results/findings** | |  |
|  | **Synthesis and interpretation** - Main findings (e.g., interpretations, inferences, and themes); might include development of a theory or model, or integration with prior research or theory | 14-27 |
|  | **Links to empirical data** - Evidence (e.g., quotes, field notes, text excerpts, photographs) to substantiate analytic findings | SI A; S1 B |
|  |  |  |
| **Discussion** | |  |
|  | **Integration with prior work, implications, transferability, and contribution(s) to the field -** Short summary of main findings; explanation of how findings and conclusions connect to, support, elaborate on, or challenge conclusions of earlier scholarship; discussion of scope of application/generalizability; identification of unique contribution(s) to scholarship in a discipline or field | 28-35 |
|  | **Limitations** - Trustworthiness and limitations of findings | 33 |
|  |  |  |
| **Other** | |  |
|  | **Conflicts of interest** - Potential sources of influence or perceived influence on study conduct and conclusions; how these were managed | N/a: Provided on article submission |
|  | **Funding** - Sources of funding and other support; role of funders in data collection, interpretation, and reporting | N/a: Provided on article submission |
