# Supplementary material for: A qualitative exploration of migraine in students attending Irish Universities
Source: PLoS One. 2024 Aug 23;19(8):e0305643. doi: 10.1371/journal.pone.0305643 (PMC11343468; doi:10.1371/journal.pone.0305643)
Supplement: Attachment — Submitted filename: In the abstract.docx [file pone.0305643.s004.docx]

In the abstract: The authors should remove ethical considerations and mention the tools used in the Methods section. In the Results section, they should highlight important findings.

How did the authors diagnose migraine and exclude other types of headaches?

In the Methods, authors should detail their questionnaire sections, including demographic data (age, gender, etc.), medication,..etc
